# Burden of chronic low back pain: Association with pain severity and prescription medication use in five large European countries

**DOI:** 10.1111/papr.13093

**Published:** 2021-11-26

**Authors:** Serge Perrot, Michael J. Doane, Dena H. Jaffe, Erika Dragon, Lucy Abraham, Lars Viktrup, Andrew G. Bushmakin, Joseph C. Cappelleri, Philip G. Conaghan

**Affiliations:** ^1^ Centre d'Evaluation et Traitement de la Douleur INSERM U987 Hôpital Cochin Université de Paris Paris France; ^2^ Kantar Horsham Pennsylvania USA; ^3^ Kantar Tel Aviv Israel; ^4^ Global Medical Affairs Pfizer Ltd Budapest Hungary; ^5^ Patient and Health Impact Pfizer Ltd Tadworth UK; ^6^ Global Product Development Eli Lilly and Company Indianapolis Indiana USA; ^7^ Pfizer Inc. Groton Connecticut USA; ^8^ Leeds Institute of Rheumatic and Musculoskeletal Medicine University of Leeds Leeds UK; ^9^ NIHR Leeds Biomedical Research Centre Leeds UK

**Keywords:** activity impairment, chronic low back pain, Europe, healthcare resource use, health‐related quality of life, work productivity impairment

## Abstract

**Objective:**

This study assessed associations between severity of, and prescription medication use for, chronic low back pain (CLBP) and health‐related quality of life, health status, work productivity, and healthcare resource utilization.

**Methods:**

This cross‐sectional study utilized SF‐12, EQ‐5D‐5L, and work productivity and activity impairment (WPAI) questionnaires, and visits to healthcare providers among adults with self‐reported CLBP participating in the National Health and Wellness Survey in Germany, France, UK, Italy, and Spain. Respondents were stratified into four groups according to pain severity (mild or moderate/severe) and prescription medication use (Rx‐treated or Rx‐untreated). Differences between groups were estimated using generalized linear models controlling for sociodemographics and health characteristics.

**Results:**

Of 2086 respondents with CLBP, 683 had mild pain (276 Rx‐untreated, 407 Rx‐treated) and 1403 had moderate/severe pain (781 Rx‐untreated, 622 Rx‐treated). Respondents with moderate/severe pain had significantly worse health‐related quality of life (SF‐12v2 physical component summary), health status (EQ‐5D‐5L), and both absenteeism and presenteeism compared with those with mild pain, including Rx‐untreated (moderate/severe pain Rx‐untreated vs. mild pain Rx‐untreated, *p* ≤ 0.05) and Rx‐treated (moderate/severe pain Rx‐treated vs. mild pain Rx‐treated, *p* ≤ 0.05) groups. Significantly more visits to healthcare providers in the last 6 months were reported for moderate/severe pain compared with mild pain for Rx‐treated (least squares mean 13.01 vs. 10.93, *p* = 0.012) but not Rx‐untreated (8.72 vs. 7.61, *p* = 0.072) groups. Health‐related quality of life (SF‐12v2 physical component summary) and health status (EQ‐5D‐5L), as well as absenteeism and presenteeism, were significantly worse, and healthcare utilization was significantly higher, in the moderate/severe pain Rx‐treated group compared with all other groups (all *p* ≤ 0.05).

**Conclusion:**

Greater severity of CLBP was associated with worse health‐related quality of life, health status, and absenteeism and presenteeism, irrespective of prescription medication use. Greater severity of CLBP was associated with increased healthcare utilization in prescription medication users.

**Key Points**

- A cross‐sectional study in Germany, France, UK, Italy, and Spain on chronic low back pain has demonstrated that:
- Greater severity of chronic low back pain is associated with worse health‐related quality of life, health status, and absenteeism and presenteeism, irrespective of prescription medication use.
- Greater severity of chronic low back pain is associated with increased healthcare utilization in prescription medication users.

## INTRODUCTION

While multiple etiologies have been characterized as responsible for causing low back pain,^1^, ^2^ most people do not have a specific cause identified for their symptoms.^3^ Low back pain may fluctuate^4^ but when persisting for longer than 3 months it is defined as chronic low back pain (CLBP).^5^, ^6^, ^7^ Biological, psychological, and social factors may contribute to this chronic primary pain condition.^7^, ^8^, ^9^ Globally, approximately 20% of people have CLBP^5^, ^10^ and low back pain is a leading cause of disability.^11^

Clinical management of CLBP is challenging. Guidelines are moving away from a focus on pharmacologic treatments given the limited efficacy and/or adverse effects of the currently available options.^6^, ^12^ A systematic review of pharmacologic therapies for CLBP found a lack of evidence for acetaminophen (and it is known to be ineffective for acute low back pain), smaller benefits than previously observed for nonsteroidal anti‐inflammatory drugs, and only modest effects for opioids in short‐term trials.^13^ Consequently, the most recent guidelines do not recommend acetaminophen for CLBP,^6^ the risk of adverse events must be considered for nonsteroidal anti‐inflammatory drugs^6^ (which may restrict their use to the lowest dose for the shortest duration^14^), and the use of weak opioids is discouraged due to the small benefits and the risk of dependence.^6^

The impact of CLBP on quality of life is considerable and the economic burden is large.^15^, ^16^, ^17^ CLBP has a detrimental effect on both physical and mental health^15^ is associated with early retirement,^17^ and can double total healthcare costs.^16^ Understanding the differential impacts of pain severity and pharmacotherapies on individual and societal burden is important to improve the management of CLBP. The current study examined health‐related quality of life, health status, work productivity and activity impairment, and healthcare resource utilization, based on pain severity and prescription medication use, in people with CLBP across five European countries.

## METHODS

This retrospective observational study analyzed pooled data from the 2016 and 2017 National Health and Wellness Survey (NHWS), for five European countries: Germany, France, UK, Italy, and Spain.

### NHWS database

The NHWS is a self‐administered, cross‐sectional, internet‐based survey of the health of a general population of over 1.2 million adults (≥18 years of age), the respondents being identified primarily through opt‐in online survey panels. The questionnaire included a base component (assessing sociodemographics, health characteristics, and diseases experienced/diagnosed), various disease‐specific modules, and a module specific to pain. The Pearl Institutional Review Board reviewed the 2016 and 2017 NHWS and determined that they meet the exemption requirements under 45CFR46.101(b)(2), and all respondents provided informed consent. The survey was translated as appropriate for each country. Stratified random sampling, based on sex and age, was used to ensure the demographic composition of the sample was representative of the adult population for each of the five countries.^18^

### Study population

The current analyses included NHWS respondents who self‐reported they had received a physician's diagnosis of lower back pain, and had experienced pain during the prior month, with pain in the past that had lasted for at least 3 months. The current cohort included all those with CLBP who participated in the pain module: in the 2016 NHWS, a probability method was utilized for inclusion into specific disease modules (to reduce respondent burden) and all those with CLBP who were randomly selected to enter into the pain module were identified; while in the 2017 NHWS, all respondents participated in the pain module. Those who reported neuropathic or phantom limb pains were excluded. Since it was possible for a respondent to complete more than one survey during the 2 years, only the most recent data for each respondent were used.

### Assessments and outcomes

Respondents were asked three questions about pain: “What is the level of severity of your pain when using medication,” “What is the level of severity of your pain when not using medication?” and “How severe is your pain?” Possible responses were mild, moderate, severe, or do not know: The maximum severity of any answer was used to categorize the respondent's current pain severity as mild, moderate, or severe. In addition, the Short Form‐McGill Pain Questionnaire (SF‐MPQ) was used to assess pain during the last week (scored from 0 = no pain to 45 = maximum pain).^19^ Categorization as prescription medication users or non‐users was based on questions relating to the current use of prescription medications for CLBP (“Which of your conditions listed below do you currently use a prescription medication to treat?” “Earlier, you indicated that you currently take a prescription medication for your pain. Please indicate which of the following prescription medications you currently use to treat your pain,” and “You indicated you use the following prescription medications. Which type of pain do you treat with each prescription medication?”).

Health‐related quality of life was assessed using the Medical Outcomes Study 12‐Item Short Form survey instrument version 2 (SF‐12v2).^20^ This 12‐item, multipurpose, generic health status instrument reports on eight domains (physical functioning, physical role limitations, bodily pain, general health, vitality, social functioning, emotional role limitations, and mental health), with physical component summary and mental component summary scores derived from these, and higher scores indicating better health‐related quality of life. Improvements of 3.29 in SF‐12 physical component summary and 3.77 in mental component summary have been reported to be clinically relevant in patients with subacute and chronic low back pain.^21^

Health status was assessed using SF‐6D (Short Form‐6 Dimensions) and EQ‐5D‐5L. The SF‐6D utility score^22^ was derived from SF‐12v2 domains, with higher score indicating better health status (from 0.3 = worst health state to 1 = best health state). The EQ‐5D‐5L^23^ uses responses on five dimensions (mobility, self‐care, usual activities, pain/discomfort, and anxiety/depression), with a higher score indicating better health status (scored from –0.59 = worse than dead to 1.0 = full health). In addition, respondents indicated their self‐rated health state on the EQ visual analog scale (EQ VAS), with a higher score indicating better health status (scored from 0 = worst imaginable health state to 100 = best imaginable health state). Minimum clinically important differences for SF‐6D (0.041)^24^ and EQ‐5D‐5L (generally in the range 0.03–0.10)^25^, ^26^, ^27^, ^28^ have been reported for various patient populations.

Work productivity and activity impairment were assessed using the general health version of the questionnaire (WPAI‐GH), a 6‐item instrument consisting of four metrics: absenteeism (the percentage of work time missed because of one's health in the past 7 days), presenteeism (the percentage of impairment experienced while at work in the past 7 days because of one's health), overall work productivity loss (an overall impairment estimate that is a combination of absenteeism and presenteeism), and activity impairment (the percentage of impairment in daily activities because of one's health in the past 7 days).^29^ Higher scores indicate worse impairment. Only respondents who reported being full‐time or part‐time employed provided data for absenteeism, presenteeism, and overall work impairment, whereas all respondents completed the activity impairment question.

Healthcare resource utilization was based on the self‐reported number of visits to healthcare providers (including primary care and specialists) and the numbers of emergency room or urgent care visits and hospitalizations in the last 6 months.

All measures and diagnoses were self‐reported, and respondents were allowed to answer “don't know” for some questions.

### Statistical analyses

The main analysis used data pooled across the five European countries. Supplemental analyses were conducted for the individual countries.

To explore differences related to pain severity and prescription medication use, respondents were categorized into four groups: (1) mild pain untreated with prescription medication (mild pain Rx‐untreated); (2) mild pain treated with prescription medication (mild pain Rx‐treated); (3) moderate/severe pain untreated with prescription medication (moderate/severe pain Rx‐untreated); and (4) moderate/severe pain treated with prescription medication (moderate/severe pain Rx‐treated). Moderate pain and severe pain were combined for analysis due to sample size considerations for country‐level analyses.

Sociodemographic data collected during the survey included age, sex, marital status, income, education, and employment status, and health characteristic data included body mass index, smoking status, alcohol consumption, exercise behavior, and self‐reported physician diagnosis within the last 12 months of anxiety, depression, insomnia or sleep disturbance. Comorbid burden was assessed using the Charlson Comorbidity Index.^30^ Bivariate analyses of sociodemographics and health characteristics across the four groups were conducted using chi‐square (categorical variables) or analysis of variance (continuous variables).^31^

Regression modeling using generalized linear models, specifying a normal distribution and identity function (SF‐12v2, SF‐6D utility score, EQ‐5D‐5L, and EQ VAS) or negative binomial distribution and log‐link function (work productivity and activity impairment and healthcare resource utilization) as appropriate,^32^ was used to estimate differences across groups, controlling for age, sex and country, and other sociodemographic and health characteristic covariates identified as being significantly different in bivariate analyses.

Complete data were available for all items except those allowing a “don't know” response. In such cases, if those variables were included as covariates in multivariable models or as outcome measures in bivariate analysis, missing values were included as a separate, defined category, or assimilated into another category or omitted altogether (depending on whether either approach was conceptually interpretable [e.g., mean differences on a continuous measure] or necessary [e.g., due to problems with model convergence]). If those variables were analyzed as outcomes (e.g., work productivity was only assessed for employed respondents), respondents with missing data were excluded from analysis (and the subsample for analysis was reported).

Statistical analyses were conducted using SPSS v23.0 or later, with *p* ≤ 0.05 considered statistically significant. No correction was made for multiple testing.

## RESULTS

Data from 2086 survey respondents with CLBP were included: 39.69% (828/2086) in Germany, 24.93% (520/2086) in France, 18.65% (389/2086) in the UK, 9.20% (192/2086) in Italy, and 7.53% (157/2086) in Spain. Of these, 53.88% (1124/2086) were younger than 60 years of age and 61.22% (1277/2086) were female (Table 1); 32.02% (668/2086) were overweight (BMI ≥25.0 and <30.0 kg/m^2^) and 30.39% (634/2086) were obese (BMI ≥30.0 kg/m^2^; Table 2). One third were categorized as having mild pain (32.74%, 683/2086) and two thirds as having moderate/severe pain (67.26%, 1403/2086). A total of 50.67% (1057/2086) of respondents reported they did not use prescription medication for CLBP, including 40.41% (276/683) of those with mild pain (mild pain Rx‐untreated) and 55.67% (781/1403) of those with moderate/severe pain (moderate/severe pain Rx‐untreated). A total of 49.33% (1029/2086) of respondents reported prescription medication use for CLBP, including 59.59% (407/683) of those with mild pain (mild pain Rx‐treated) and 44.33% (622/1403) of those with moderate/severe pain (moderate/severe pain Rx‐treated).

**TABLE 1:** Sociodemographics of respondents with chronic low back pain

	Total (*n* = 2086)	Mild pain Rx‐untreated (*n* = 276)	Mild pain Rx‐treated (*n* = 407)	Moderate/severe pain Rx‐untreated (*n* = 781)	Moderate/severe pain Rx‐treated (*n* = 622)	*p* Value^a^
Country, *n* (%)						
Germany	828 (39.69)	116 (42.03)	149 (36.61)	312 (39.95)	251 (40.35)	<0.001
France	520 (24.93)	40 (14.49)	98 (24.08)	209 (26.76)	173 (27.81)	
United Kingdom	389 (18.65)	70 (25.36)	79 (19.41)	116 (14.85)	124 (19.94)	
Italy	192 (9.20)	28 (10.14)	32 (7.86)	100 (12.80)	32 (5.14)	
Spain	157 (7.53)	22 (7.97)	49 (12.04)	44 (5.63)	42 (6.75)	
Age, years, mean (SD)	56.35 (13.22)	54.86 (13.86)	57.63 (12.65)	54.52 (14.23)	58.45 (11.48)	<0.001
Age groups, years, *n* (%)						
18–39	247 (11.84)	34 (12.32)	39 (9.58)	134 (17.16)	40 (6.43)	<0.001
40–49	347 (16.63)	57 (20.65)	67 (16.46)	130 (16.65)	93 (14.95)	
50–59	530 (25.41)	72 (26.09)	98 (24.08)	180 (23.05)	180 (28.94)	
60–69	646 (30.97)	73 (26.45)	134 (32.92)	234 (29.96)	205 (32.96)	
≥70	316 (15.15)	40 (14.49)	69 (16.95)	103 (13.19)	104 (16.72)	
Female, *n* (%)	1277 (61.22)	149 (53.99)	250 (61.43)	493 (63.12)	385 (61.90)	0.060
Marital status, *n* (%)						
Married/living with partner	1279 (61.31)	170 (61.59)	261 (64.13)	474 (60.69)	374 (60.13)	0.002
Single	352 (16.87)	59 (21.38)	64 (15.72)	147 (18.82)	82 (13.18)	
Divorced/separated/widowed	453 (21.72)	47 (17.03)	82 (20.15)	160 (20.49)	164 (26.37)	
Declined to answer	2 (0.10)	0	0	0	2 (0.32)	
Household income, *n* (%)						
<€50K/£40K per year	1579 (75.70)	190 (68.84)	320 (78.62)	572 (73.24)	497 (79.90)	<0.001
≥€50K/£40K per year	356 (17.07)	66 (23.91)	65 (15.97)	154 (19.72)	71 (11.41)	
Declined to answer	151 (7.24)	20 (7.25)	22 (5.41)	55 (7.04)	54 (8.68)	
Education, *n* (%)						
Completed university	534 (25.60)	89 (32.25)	93 (22.85)	222 (28.43)	130 (20.90)	0.002
Not completed university	1535 (73.59)	183 (66.30)	311 (76.41)	555 (71.06)	486 (78.14)	
Declined to answer	17 (0.81)	4 (1.45)	3 (0.74)	4 (0.51)	6 (0.96)	
Employed, *n* (%)^b^	841 (40.32)	142 (51.45)	158 (38.82)	346 (44.30)	195 (31.35)	<0.001

Abbreviations: Rx, prescription medication; SD, standard deviation.

^a^ Bivariate analyses, chi‐square (categorical variables) or analysis of variance (continuous variables).

^b^ Full‐time, part‐time, or self‐employed. Not employed included: not employed, disabled, retired, student, or homemaker.

**TABLE 2:** Health characteristics of respondents with chronic low back pain

	Total (*n* = 2086)	Mild pain Rx‐untreated (*n* = 276)	Mild pain Rx‐treated (*n* = 407)	Moderate/severe pain Rx‐untreated (*n* = 781)	Moderate/severe pain Rx‐treated (*n* = 622)	*p* Value^a^
Body mass index, *n* (%)						
Under/normal weight (<25.0 kg/m^2^)	705 (33.80)	105 (38.04)	128 (31.45)	298 (38.16)	174 (27.97)	<0.001
Overweight (≥25.0 and <30.0 kg/m^2^)	668 (32.02)	99 (35.87)	139 (34.15)	240 (30.73)	190 (30.55)	
Obese (≥30.0 kg/m^2^)	634 (30.39)	68 (24.64)	123 (30.22)	214 (27.40)	229 (36.82)	
Declined to answer	79 (3.79)	4 (1.45)	17 (4.18)	29 (3.71)	29 (4.66)	
Smoking status, *n* (%)						
Current	617 (29.58)	69 (25.00)	115 (28.26)	220 (28.17)	213 (34.24)	0.025
Former	748 (35.86)	95 (34.42)	150 (36.86)	281 (35.98)	222 (35.69)	
Never	721 (34.56)	112 (40.58)	142 (34.89)	280 (35.85)	187 (30.06)	
Alcohol status, *n* (%)						
None or low (≤once a month)	969 (46.45)	107 (38.77)	179 (43.98)	337 (43.15)	346 (55.63)	<0.001
Moderate (2–3 times per week up to 2–3 times per month)	820 (39.31)	116 (42.03)	174 (42.75)	323 (41.36)	207 (33.28)	
High (≥4 drinks per day up to ≥4 drinks per week)	297 (14.24)	53 (19.20)	54 (13.27)	121 (15.49)	69 (11.09)	
Exercise status						
Exercise, *n* (%)^b^	1166 (55.90)	188 (68.12)	224 (55.04)	471 (60.31)	283 (45.50)	<0.001
No exercise, *n* (%)^b^	920 (44.10)	88 (31.88)	183 (44.96)	310 (39.69)	339 (54.50)	
Exercised vigorously, days per month, mean (SD)^c^	6.14 (7.98)	7.48 (7.91)	5.84 (7.72)	6.53 (8.03)	5.24 (8.02)	<0.001
CCI score, *n* (%)^d^						
0	1217 (58.34)	203 (73.55)	215 (52.83)	504 (64.53)	295 (47.43)	<0.001
1	498 (23.87)	49 (17.75)	102 (25.06)	179 (22.92)	168 (27.01)	
≥2	371 (17.79)	24 (8.70)	90 (22.11)	98 (12.55)	159 (25.56)	
Anxiety, *n* (%)^e^	497 (23.83)	33 (11.96)	122 (29.98)	167 (21.38)	175 (28.14)	<0.001
Depression, *n* (%)^e^	515 (24.69)	40 (14.49)	109 (26.78)	167 (21.38)	199 (31.99)	<0.001
Sleep disturbance (e.g., insomnia, sleep apnea), *n* (%)^e^	502 (24.07)	32 (11.59)	109 (26.78)	158 (20.23)	203 (32.64)	<0.001
Pain^f^						
SF‐MPQ, mean (SD)	13.60 (9.44)	6.41 (5.61)	13.17 (8.11)	11.79 (8.35)	19.34 (9.71)	<0.001

Abbreviations: CCI, Charlson Comorbidity Index; Rx, prescription medication; SD, standard deviation; SF‐MPQ, Short Form‐McGill Pain Questionnaire.

^a^ Bivariate analyses, chi‐square (categorical variables) or analysis of variance (continuous variables).

^b^ Exercised for at least 20 min at least once in past month.

^c^ The question was: “How many days in the past month did you exercise vigorously for at least 20 min for the purpose of improving or maintaining your health, with the purpose of losing weight, or for enjoyment?”

^d^ CCI calculated by weighting and summing the presence of: HIV/AIDS, metastatic tumor, lymphoma, leukemia, any tumor, moderate/severe renal disease, hemiplegia or paraplegia, diabetes, diabetes with end organ damage, mild or moderate/severe liver disease, ulcer disease, connective tissue disease, chronic pulmonary disease, dementia, cerebrovascular disease, peripheral vascular disease, myocardial infarction, and congestive heart failure.^30^ The CCI in the survey did not include moderate/severe liver disease or paraplegia, as those conditions were not assessed. A higher CCI index score indicates a greater comorbid burden on the individual.

^e^ Self‐reported physician diagnosis in past 12 months.

^f^ The question asked was: “Please describe your pain during the last week.” The SF‐MPQ evaluates the sensory, affective, and evaluative dimensions of the pain experience by assessing the severity and type of pain using 15 descriptors (11 sensory, 4 affective, each rated: 0 = none, 1 = mild, 2 = moderate, 3 = severe) with total score ranging from 0 = no pain to 45 = maximum pain.^19^

Bivariate analyses identified significant differences in sociodemographics (Table 1) and health characteristics (Table 2) across the four groups, including age, marital status, education, income, employment status, alcohol use, exercise, body mass index, smoking status, Charlson Comorbidity Index, and diagnoses of anxiety, depression, insomnia or sleep difficulties; these covariates, as well as sex and country of residence, were included in multivariate analyses. In the moderate/severe pain groups, there were more obese patients, smokers and those living with comorbidities, and fewer patients exercised or drank alcohol (moderate or high consumption), than in the mild pain groups (Table 2). Total SF‐MPQ scores (mean) were 6.41 (mild pain Rx‐untreated), 13.17 (mild pain Rx‐treated), 11.79 (moderate/severe pain Rx‐untreated), and 19.34 (moderate/severe pain Rx‐treated) (*p* < 0.001; Table 2). All four groups consulted a wide variety of healthcare professionals and reported over‐the‐counter medication use, and 55.59% (572/1029) of prescription medication‐treated respondents reported opioid use (Table 3).

**TABLE 3:** Medication use and healthcare seeking by respondents with chronic low back pain

	Total (*n* = 2086)	Mild pain Rx‐untreated (*n* = 276)	Mild pain Rx‐treated (*n* = 407)	Moderate/severe pain Rx‐untreated (*n* = 781)	Moderate/severe pain Rx‐treated (*n* = 622)	*p* Value^a^
Prescription medication use (yes), *n* (%)						
NSAID	—	—	275 (67.57)	—	397 (63.83)	0.228
Opioid	—	—	185 (45.45)	—	387 (62.22)	<0.001
Acetaminophen	—	—	125 (30.71)	—	179 (28.78)	0.530
Anticonvulsant	—	—	45 (11.06)	—	105 (16.88)	0.011
Muscle relaxant	—	—	8 (1.97)	—	11 (1.77)	1.000
Topical anesthetic	—	—	6 (1.47)	—	10 (1.61)	1.000
DMARD	—	—	2 (0.49)	—	4 (0.64)	1.000
Biologic	—	—	1 (0.25)	—	3 (0.48)	0.656
Steroid	—	—	1 (0.25)	—	3 (0.48)	0.656
Triptan	—	—	7 (1.72)	—	3 (0.48)	0.057
Analgesic	—	—	1 (0.25)	—	1 (0.16)	1.000
Other	—	—	59 (14.50)	—	91 (14.63)	1.000
Maximum number of days of CLBP medication used during prior month, mean (SD)^b^	18.65 (11.12)	—	15.72 (10.76)	—	20.50 (10.96)	<0.001
Duration of taking medications for CLBP, months, mean (SD)^b^	121.28 (116.22)	—	120.06 (122.15)	—	122.04 (112.42)	0.798
Non‐prescription medication use (yes), *n* (%)^c^	835 (40.03)	135 (48.91)	115 (28.26)	454 (58.13)	131 (21.06)	<0.001
Visited healthcare provider (traditional) in last 6 months (yes), *n* (%)						
Emergency room or urgent care	314 (15.05)	17 (6.16)	75 (18.43)	105 (13.44)	117 (18.81)	<0.001
Hospitalization	273 (13.09)	14 (5.07)	61 (14.99)	79 (10.12)	119 (19.13)	<0.001
Other	2034 (97.51)	259 (93.84)	404 (99.26)	757 (96.93)	614 (98.71)	<0.001
Primary care	1819 (87.20)	215 (77.90)	371 (91.15)	659 (84.38)	574 (92.28)	<0.001
Visited practitioner (yes), *n* (%)						
Acupuncturist	—	11 (3.99)	14 (3.44)	33 (4.23)	45 (7.23)	0.016
Chiropractor	—	8 (2.90)	13 (3.19)	34 (4.35)	28 (4.50)	0.526
Herbalist	—	6 (2.17)	7 (1.72)	31 (3.97)	16 (2.57)	0.794
Physical therapist	—	50 (18.12)	96 (23.59)	153 (19.59)	128 (20.58)	0.290
Nutritionist	—	3 (1.09)	13 (3.19)	15 (1.92)	11 (1.77)	0.231
Massage therapist	—	30 (10.87)	67 (16.46)	112 (14.34)	148 (23.79)	<0.001
Occupational therapist	—	0	10 (2.46)	5 (0.64)	15 (2.41)	0.002
Pharmacist	—	134 (48.55)	241 (59.21)	433 (55.44)	395 (63.50)	<0.001
Pharmacy assistant	—	37 (13.41)	51 (12.53)	80 (10.24)	73 (11.74)	0.451
Homeopath	—	5 (4.31)	5 (3.36)	18 (5.77)	16 (6.37)	0.561

Abbreviations: CLBP, chronic low back pain; DMARD, disease‐modifying anti‐rheumatic drug; NSAID, nonsteroidal anti‐inflammatory drug; Rx, prescription medication; SD, standard deviation.

^a^ Bivariate analyses, chi‐square (categorical variables) or analysis of variance (continuous variables).

^b^ Sample size: total (*n* = 957), mild pain Rx‐untreated (*n* = 0), mild pain Rx‐treated (*n* = 370), moderate/severe pain Rx‐untreated (*n* = 0), moderate/severe pain Rx‐treated (*n* = 587).

^c^ In response to the question, “Do you use a non‐prescription medication (e.g., over‐the‐counter medication, thermacare, wraps) or herbal product to treat your pain?”

**FIGURE 1:**
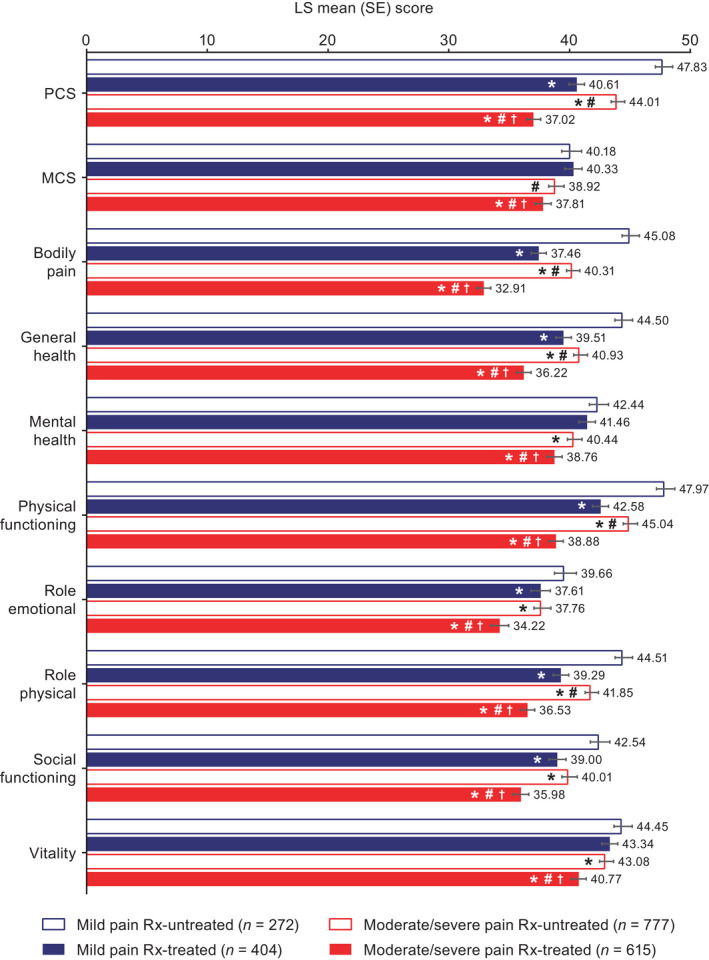
Health‐related quality of life of respondents with chronic low back pain: SF‐12v2. *Differs from mild pain Rx‐untreated, *p* ≤ 0.05. ^#^Differs from mild pain Rx‐treated, *p* ≤ 0.05. ^†^Differs from moderate/severe pain Rx‐untreated, *p* ≤ 0.05. Higher scores indicate a better quality of life. Generalized linear models specifying a normal distribution and identity function were used to assess differences in health‐related quality of life by group. PCS and MCS scores are normed to a mean of 50 and a standard deviation of 10 for the US population. Covariates included: severity/treatment group, country of residence, age, sex, marital status, education, income, employment status, alcohol use, exercise, body mass index, smoking status, anxiety diagnosis, depression diagnosis, insomnia diagnosis, diagnosed with sleep difficulties, and CCI. A total of 18 respondents had missing data and were excluded from multivariate analyses. CCI, Charlson Comorbidity Index; LS, least squares; MCS, mental component summary score; PCS, physical component summary score; Rx, prescription medication; SE, standard error; SF‐12v2, Medical Outcomes Study 12‐Item Short Form Survey Instrument version 2

**FIGURE 2:**
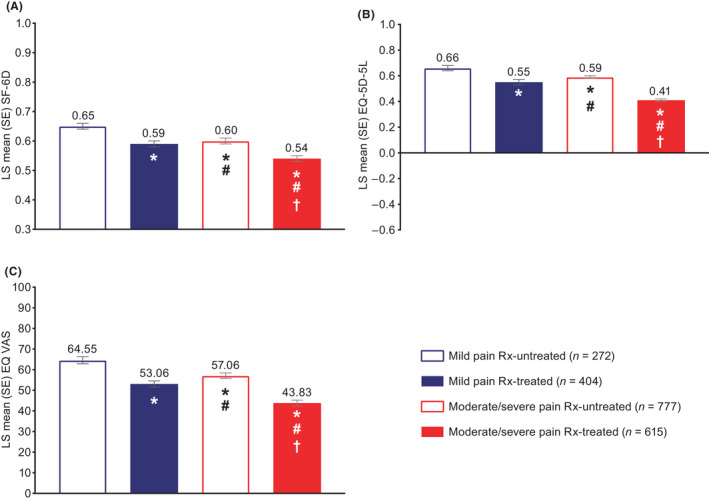
Health status of respondents with chronic low back pain: (A) SF‐6D utility score, (B) EQ‐5D‐5L index value, and (C) EQ VAS. *Differs from mild pain Rx‐untreated, *p* ≤ 0.05. ^#^Differs from mild pain Rx‐treated, *p* ≤ 0.05. ^†^Differs from moderate/severe pain Rx‐untreated, *p* ≤ 0.05. Higher scores indicate better health status. The SF‐6D index has interval scoring properties and yields summary scores from 0.3 (worst health state) to 1 (best health state). The EQ‐5D‐5L ranges from −0.59 (where 0 is the value of a health state equivalent to dead, and negative values represent values as worse than dead) to 1 (the value of full health). EQ‐5D‐5L scoring used crosswalk mapping the 5L dimension scores onto the 3L value sets,^42^, ^43^, ^44^ and the UK preference‐based set of utilities (1 to −0.594) was used for all countries based on the publisher's recommendation. The EQ VAS ranges from 0 (worst imaginable health state) to 100 (best imaginable health state). Generalized linear models specifying a normal distribution and identity function assessed differences in health status by group. Covariates included: severity/treatment group, country of residence, age, sex, marital status, education, income, employment status, alcohol use, exercise, body mass index, smoking status, anxiety diagnosis, depression diagnosis, insomnia diagnosis, diagnosed with sleep difficulties, and CCI. A total of 18 respondents had missing data and were excluded from multivariate analyses. CCI, Charlson Comorbidity Index; EQ VAS, EQ visual analog scale; LS, least squares; Rx, prescription medication; SE, standard error; SF‐6D, Short Form‐6 Dimensions

**FIGURE 3:**
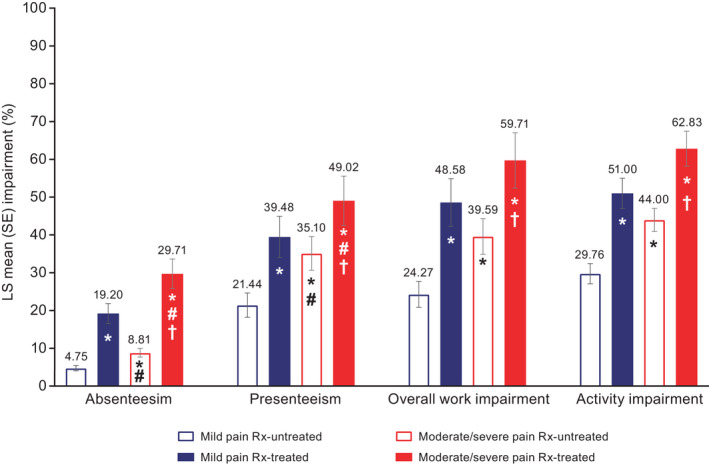
Work productivity and activity impairment among respondents with chronic low back pain: WPAI‐GH. *Differs from mild pain Rx‐untreated, *p* ≤ 0.05. ^#^Differs from mild pain Rx‐treated, *p* ≤ 0.05. ^†^Differs from moderate/severe pain Rx‐untreated, *p* ≤ 0.05. Sample sizes for mild pain Rx‐untreated, mild pain Rx‐treated, moderate/severe pain Rx‐untreated, and moderate/severe pain Rx‐treated groups, respectively: absenteeism (*n* = 135, *n* = 141, *n* = 320, *n* = 191), presenteeism (*n* = 132, *n* = 128, *n* = 303, *n* = 162), overall work impairment (*n* = 135, *n* = 141, *n* = 320, *n* = 191), and activity impairment (*n* = 272, *n* = 404, *n* = 777, *n* = 615). Higher scores indicate greater impairment (worse outcome). Generalized linear models specifying a negative binomial distribution and log‐link function assessed differences in work and activity impairment by group. Covariates included: severity/treatment group, country of residence, age, sex, marital status, education, income, employment status, alcohol use, exercise, body mass index, smoking status, anxiety diagnosis, depression diagnosis, insomnia diagnosis, diagnosed with sleep difficulties, and CCI. A total of 18 respondents had missing data and were excluded from multivariate analyses. Only respondents who reported being full‐time or part‐time employed provided data for absenteeism, presenteeism, and overall work impairment, whereas all respondents completed the activity impairment question. CCI, Charlson Comorbidity Index; LS, least squares; Rx, prescription; SE, standard error; WPAI‐GH, Work Productivity and Activity Impairment‐General Health

**FIGURE 4:**
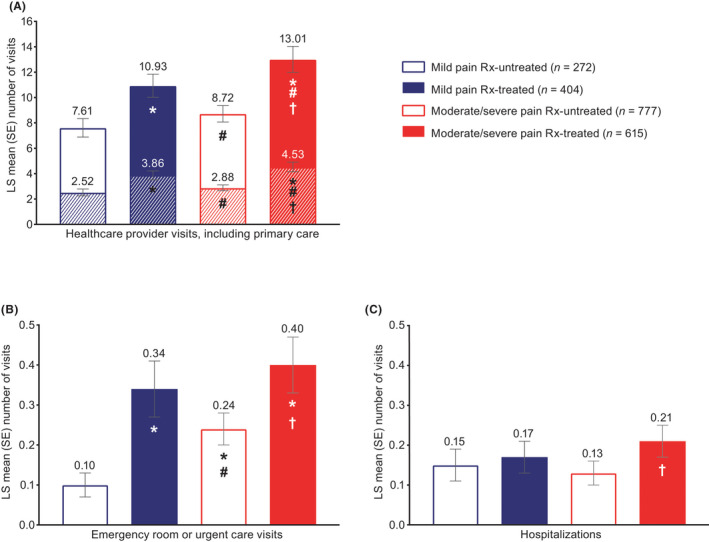
Healthcare resource utilization in the past 6 months among respondents with chronic low back pain: number of (A) healthcare provider visits including primary care, (B) emergency room or urgent care visits, and (C) hospitalizations. *Differs from mild pain Rx‐untreated, *p* ≤ 0.05. ^#^Differs from mild pain Rx‐treated, *p* ≤ 0.05. ^†^Differs from moderate/severe pain Rx‐untreated, *p* ≤ 0.05. Higher number of visits indicates more healthcare utilization. Panel A shows the contribution of primary care visits (hashed area) to healthcare provider visits (solid area). Generalized linear models specifying a negative binomial distribution and log‐link function assessed differences in healthcare resource utilization by group. Covariates included: severity/treatment group, country of residence, age, sex, marital status, education, income, employment status, alcohol use, exercise, body mass index, smoking status, anxiety diagnosis, depression diagnosis, insomnia diagnosis, diagnosed with sleep difficulties, and CCI. A total of 18 respondents had missing data and were excluded from multivariate analyses. CCI, Charlson Comorbidity Index; LS, least squares; Rx, prescription medication; SE, standard error

After controlling for sociodemographics and health characteristics, health‐related quality of life (SF‐12v2 physical component summary) was significantly worse for respondents with moderate/severe pain compared with those with mild pain, including Rx‐untreated (44.01 vs. 47.83, respectively, *p* < 0.001) and Rx‐treated (least squares mean, 37.02 vs. 40.61, respectively, *p* < 0.001) groups (Figure 1). For the mental component summary, health‐related quality of life was significantly worse for moderate/severe pain compared with mild pain for Rx‐treated (*p* < 0.001) but not Rx‐untreated (*p* = 0.057) groups (Figure 1). Health status (all measures) was significantly worse for respondents with moderate/severe pain compared with those with mild pain (Figure 2), including EQ‐5D‐5L for Rx‐untreated (0.59 vs. 0.66, respectively, *p* < 0.001) and Rx‐treated (0.41 vs. 0.55, respectively, *p* < 0.001) groups. Absenteeism was significantly worse for respondents with moderate/severe pain compared with those with mild pain, including Rx‐untreated (8.81% vs. 4.75%, respectively, *p* < 0.001) and Rx‐treated (29.71% vs. 19.20%, respectively, *p* = 0.002) groups (Figure 3). Presenteeism was significantly worse for respondents with moderate/severe pain compared with those with mild pain, including Rx‐untreated (35.10% vs. 21.44%, respectively, *p* < 0.001) and Rx‐treated (49.02% vs. 39.48%, respectively, *p* = 0.002) groups (Figure 3). Both overall work impairment and activity impairment were significantly worse for respondents with moderate/severe pain compared with those with mild pain for Rx‐untreated (both *p* < 0.001) but not Rx‐treated (*p* = 0.081 for overall work impairment and *p* = 0.119 for activity impairment) groups (Figure 3). Significantly more visits to healthcare providers in the last 6 months were reported for moderate/severe pain compared with mild pain, for Rx‐treated (13.01 moderate/severe pain Rx‐treated vs. 10.93 mild pain Rx‐treated, *p* = 0.012) but not Rx‐untreated (8.72 moderate/severe pain Rx‐untreated vs. 7.61 mild pain Rx‐untreated, *p* = 0.072) groups (Figure 4).

Health‐related quality of life (SF‐12v2 physical component summary; Figure 1), health status (all measures; Figure 2), and absenteeism and presenteeism (Figure 3) were significantly worse, and healthcare provider visits were significantly higher (Figure 4), in the moderate/severe pain Rx‐treated group compared with all other groups (all *p* ≤ 0.05). The mild pain Rx‐treated group had worse health‐related quality of life (SF‐12v2 physical component summary; Figure 1), health status (Figure 2), and absenteeism and presenteeism (Figure 3) than the moderate/severe pain Rx‐untreated group (all *p* ≤ 0.05).

Analyses of the corresponding data for respondents in Germany (Table S1), France (Table S2), UK (Table S3), Italy (Table S4), and Spain (Table S5) broadly reflected the results of the pooled data.

## DISCUSSION

This study across five European countries showed that compared with mild CLBP, moderate/severe CLBP was associated with worse health‐related quality of life, health status, and absenteeism and presenteeism, irrespective of prescription medication use, and higher resource utilization in prescription medication users.

The association between CLBP severity and increased individual and societal burden supports previous findings.^33^, ^34^, ^35^, ^36^, ^37^ The magnitude of the differences in health‐related quality of life and health status between the moderate/severe pain and mild pain groups was considerable, exceeding minimum clinically important differences for SF‐12 physical component summary,^21^ SF‐6D,^24^ and EQ‐5D‐5L.^25^, ^26^, ^27^

It is interesting to note that the mild pain Rx‐treated group had worse health‐related quality of life than the moderate/severe pain Rx‐untreated group in the current study. Although prescription medication status differed between the groups, there was also disparity between the pain categorization (as mild or moderate/severe) and the groups’ mean SF‐MPQ scores. This could reflect the different questions and assessments used: while the categorization as mild or moderate/severe related to the most severe current pain severity, the SF‐MPQ score was based on the pain experience over the previous week and on a variety of sensory and affective descriptors.

Although this was a cross‐sectional study, the current data suggest that existing pharmacological therapies for CLBP have small benefits. Despite the reported limited efficacy of opioids,^13^, ^38^, ^39^ a large proportion of the prescription medication‐treated respondents (55.59%) in the current study reported opioid use. The inadequacy of current treatments for CLBP is further reflected in a European study showing that 80% of those diagnosed with low back pain by a physician at least 6 years previously still experienced moderate or severe possible/probable chronic pain.^40^ Satisfaction with medication is lower in those with more severe CLBP.^35^ A greater proportion of the mild pain group than the moderate/severe pain group used prescription medication in the current study, and an explanation for this is not clear from the current data.

Prescription medication use was not associated with better health‐related quality of life in this study, regardless of pain severity, supporting previous findings in osteoarthritis.^41^ Given the cross‐sectional study design, this finding should be interpreted cautiously, since the methodology could not take into account respondent preferences for non‐pharmacologic approaches or over‐the‐counter medications, past history of prescription medication use, nor access to prescription medication or differences in healthcare practices across the five countries.

This cross‐sectional study had some further limitations. It is not possible to establish causality from the current data. Since the categorization of respondents was based on maximum current pain severity, the prescription medication‐treated groups could have included respondents whose pain was to some extent controlled by current medication. There was likely to be some channeling bias; for example, nonsteroidal anti‐inflammatory drugs would not have been prescribed to people who were previously intolerant or who had contraindications due to comorbidities. Although the use of stratified random sampling provides a sample that is broadly representative of the adult population for the countries surveyed, there may be selection bias with those respondents self‐reporting a CLBP diagnosis not being fully representative of the CLBP population. The data, which were self‐reported by respondents and not verified by a healthcare professional, are also subject to recall bias.

## CONCLUSION

This study showed that, compared with mild CLBP, moderate/severe CLBP was associated with worse health‐related quality of life, health status, and absenteeism and presenteeism, regardless of prescription medication use, and increased healthcare utilization in prescription medication users. There were significant differences across the four groups for multiple sociodemographic, health and treatment characteristics in these patients with CLBP. Differences were evident in each of the five countries, regardless of the various healthcare systems. These data clearly underline the limitation of current pharmacological treatments, and the need to optimize the management of CLBP to reduce the burden for the individual and society.

## CONFLICTS OF INTEREST

Serge Perrot has received fees for advisory boards and consultancy from Pfizer, Menarini, Grünenthal and UPSA, and research grants from Grünenthal. At the time the study was conducted, Michael J Doane was an employee of Kantar Health, which was paid by Pfizer and Eli Lilly and Company in connection with the research and development of this manuscript. Dena H Jaffe is an employee of Kantar, which was paid by Pfizer and Eli Lilly and Company in connection with the research and development of this manuscript. Erika Dragon is an employee of Pfizer with stock and/or stock options. Lucy Abraham is an employee of Pfizer with stock and/or stock options. Lars Viktrup is an employee of Eli Lilly and Company and owns stocks in Lilly. Andrew G Bushmakin is an employee of Pfizer with stock and/or stock options. Joseph C Cappelleri is an employee of Pfizer with stock and/or stock options. Philip G Conaghan has done consultancies or speakers bureaus for AbbVie, AstraZeneca, BMS, Centrexion, EMD Serono, Flexion Therapeutics, Galapagos, Gilead, Novartis, and Pfizer.

## AUTHOR CONTRIBUTIONS

Michael J Doane and Dena H Jaffe contributed to the conception or design of the study (with discussion from all authors) and acquisition of data. All authors contributed to the analysis or interpretation of data. All authors contributed to drafting the manuscript and revising it critically for important intellectual content. All authors approved the final version to be published and agree to be accountable for all aspects of the work in ensuring that questions related to the accuracy or integrity of any part of the work are appropriately investigated and resolved.

## DATA AVAILABILITY STATEMENT

Data collection of the NHWS was performed independently by Kantar. The data used to generate the study results will be made available upon request.

## Supporting information

Table S1‐S5

## References

[1] Borenstein DG . Chronic low back pain. Rheum Dis Clin North Am. 1996;22:439–56. 8844907 10.1016/s0889-857x(05)70281-7

[2] Perrot S , Cohen M , Barke A , Korwisi B , Rief W , Treede RD . The IASP classification of chronic pain for ICD‐11: chronic secondary musculoskeletal pain. Pain. 2019;160:77–82. 30586074 10.1097/j.pain.0000000000001389

[3] Hartvigsen J , Hancock MJ , Kongsted A , Louw Q , Ferreira ML , Genevay S , et al. What low back pain is and why we need to pay attention. Lancet. 2018;391:2356–67. 29573870 10.1016/S0140-6736(18)30480-X

[4] Chen Y , Campbell P , Strauss VY , Foster NE , Jordan KP , Dunn KM . Trajectories and predictors of the long‐term course of low back pain: cohort study with 5‐year follow‐up. Pain. 2018;159:252–60. 29112007 10.1097/j.pain.0000000000001097 PMC5771685

[5] Meucci RD , Fassa AG , Faria NM . Prevalence of chronic low back pain: systematic review. Rev Saude Publica. 2015;49:1. 26487293 10.1590/S0034-8910.2015049005874 PMC4603263

[6] Oliveira CB , Maher CG , Pinto RZ , Traeger AC , Lin C‐W , Chenot J‐F , et al. Clinical practice guidelines for the management of non‐specific low back pain in primary care: an updated overview. Eur Spine J. 2018;27:2791–803. 29971708 10.1007/s00586-018-5673-2

[7] Nicholas M , Vlaeyen JWS , Rief W , Barke A , Aziz Q , Benoliel R , et al. The IASP classification of chronic pain for ICD‐11: chronic primary pain. Pain. 2019;160:28–37. 30586068 10.1097/j.pain.0000000000001390

[8] Chou R , Shekelle P . Will this patient develop persistent disabling low back pain? JAMA. 2010;303:1295–302. 20371789 10.1001/jama.2010.344

[9] Pincus T , Burton AK , Vogel S , Field AP . A systematic review of psychological factors as predictors of chronicity/disability in prospective cohorts of low back pain. Spine (Phila Pa 1976). 2002;27:E109–20. 11880847 10.1097/00007632-200203010-00017

[10] Hoy D , Bain C , Williams G , March L , Brooks P , Blyth F , et al. A systematic review of the global prevalence of low back pain. Arthritis Rheum. 2012;64:2028–37. 22231424 10.1002/art.34347

[11] Global Burden of Disease Collaborators . Global, regional, and national incidence, prevalence, and years lived with disability for 354 diseases and injuries for 195 countries and territories, 1990–2017: a systematic analysis for the Global Burden of Disease Study 2017. Lancet. 2018;392:1789–858. 30496104 10.1016/S0140-6736(18)32279-7 PMC6227754

[12] Schreijenberg M , Koes BW , Lin CC . Guideline recommendations on the pharmacological management of non‐specific low back pain in primary care – is there a need to change? Expert Rev Clin Pharmacol. 2019;12:145–57. 30618319 10.1080/17512433.2019.1565992

[13] Chou R , Deyo R , Friedly J , Skelly A , Weimer M , Fu R , et al. Systemic pharmacologic therapies for low back pain: a systematic review for an American College of Physicians clinical practice guideline. Ann Intern Med. 2017;166:480–92. 28192790 10.7326/M16-2458

[14] National Institute for Health and Care Excellence . Low back pain and sciatica in over 16s: assessment and management. NICE guideline NG59; 2016. https://www.nice.org.uk/guidance/ng59. Accessed 24 June 2020 31841295

[15] Husky MM , Ferdous Farin F , Compagnone P , Fermanian C , Kovess‐Masfety V . Chronic back pain and its association with quality of life in a large French population survey. Health Qual Life Outcomes. 2018;16:195. 30257670 10.1186/s12955-018-1018-4 PMC6158815

[16] Hong J , Reed C , Novick D , Happich M . Costs associated with treatment of chronic low back pain: an analysis of the UK General Practice Research Database. Spine (Phila Pa 1976). 2013;38:75–82. 23038621 10.1097/BRS.0b013e318276450f

[17] Gouveia N , Rodrigues A , Eusébio M , Ramiro S , Machado P , Canhão H , et al. Prevalence and social burden of active chronic low back pain in the adult Portuguese population: results from a national survey. Rheumatol Int. 2016;36:183–97. 26661091 10.1007/s00296-015-3398-7

[18] United States Census Bureau . International data base; 2018. https://www.census.gov/programs‐surveys/international‐programs/about/idb.html. Accessed 27 August 2019

[19] Melzack R . The short‐form McGill pain questionnaire. Pain. 1987;30:191–7. 3670870 10.1016/0304-3959(87)91074-8

[20] Ware J Jr , Kosinski M , Keller SD . A 12‐Item Short‐Form Health Survey: construction of scales and preliminary tests of reliability and validity. Med Care. 1996;34:220–33. 8628042 10.1097/00005650-199603000-00003

[21] Díaz‐Arribas MJ , Fernández‐Serrano M , Royuela A , Kovacs FM , Gallego‐Izquierdo T , Ramos‐Sánchez M , et al. Minimal clinically important difference in quality of life for patients with low back pain. Spine (Phila Pa 1976). 2017;42:1908–16. 28658040 10.1097/BRS.0000000000002298

[22] Brazier JE , Roberts J . The estimation of a preference‐based measure of health from the SF‐12. Med Care. 2004;42:851–9. 15319610 10.1097/01.mlr.0000135827.18610.0d

[23] Herdman M , Gudex C , Lloyd A , Janssen MF , Kind P , Parkin D , et al. Development and preliminary testing of the new five‐level version of EQ‐5D (EQ‐5D‐5L). Qual Life Res. 2011;20:1727–36. 21479777 10.1007/s11136-011-9903-x PMC3220807

[24] Walters SJ , Brazier JE . Comparison of the minimally important difference for two health state utility measures: EQ‐5D and SF‐6D. Qual Life Res. 2005;14:1523–32. 16110932 10.1007/s11136-004-7713-0

[25] McClure NS , Sayah FA , Xie F , Luo N , Johnson JA . Instrument‐defined estimates of the minimally important difference for EQ‐5D‐5L index scores. Value Health. 2017;20:644–50. 28408007 10.1016/j.jval.2016.11.015

[26] Nolan CM , Longworth L , Lord J , Canavan JL , Jones SE , Kon SSC , et al. The EQ‐5D‐5L health status questionnaire in COPD: validity, responsiveness and minimum important difference. Thorax. 2016;71:493–500. 27030578 10.1136/thoraxjnl-2015-207782 PMC4893131

[27] McClure NS , Sayah FA , Ohinmaa A , Johnson JA . Minimally important difference of the EQ‐5D‐5L index score in adults with type 2 diabetes. Value Health. 2018;21:1090–7. 30224114 10.1016/j.jval.2018.02.007

[28] Chen P , Lin KC , Liing RJ , Wu CY , Chen CL , Chang KC . Validity, responsiveness, and minimal clinically important difference of EQ‐5D‐5L in stroke patients undergoing rehabilitation. Qual Life Res. 2016;25:1585–96. 26714699 10.1007/s11136-015-1196-z

[29] Reilly MC , Zbrozek AS , Dukes EM . The validity and reproducibility of a work productivity and activity impairment instrument. Pharmacoeconomics. 1993;4:353–65. 10146874 10.2165/00019053-199304050-00006

[30] Charlson ME , Pompei P , Ales KL , MacKenzie CR . A new method of classifying prognostic comorbidity in longitudinal studies: development and validation. J Chronic Dis. 1987;40:373–83. 3558716 10.1016/0021-9681(87)90171-8

[31] van Belle G , Fisher LD , Heagerty PJ , Lumley T . Biostatistics: a methodology for the health sciences. 2nd ed. Hoboken: John Wiley & Sons; 2004.

[32] Agresti A . Foundations of linear and generalized linear models. 1st ed. Hoboken: John Wiley & Sons; 2015.

[33] Montgomery W , Vietri J , Shi J , et al. The relationship between pain severity and patient‐reported outcomes among patients with chronic low back pain in Japan. J Pain Res. 2016;9:337–44. 27330326 10.2147/JPR.S102063 PMC4898257

[34] Mutubuki EN , Beljon Y , Maas ET , Huygen FJPM , Ostelo RWJG , van Tulder MW , et al. The longitudinal relationships between pain severity and disability versus health‐related quality of life and costs among chronic low back pain patients. Qual Life Res. 2020;29:275–87. 31531837 10.1007/s11136-019-02302-w PMC6962124

[35] Sadosky AB , Taylor‐Stokes G , Lobosco S , Pike J , Ross E . Relationship between self‐reported low‐back pain severity and other patient‐reported outcomes: results from an observational study. J Spinal Disord Tech. 2013;26:8–14. 21909037 10.1097/BSD.0b013e3182296c15

[36] Depont F , Hunsche E , Abouelfath A , Diatta T , Addra I , Grelaud A , et al. Medical and non‐medical direct costs of chronic low back pain in patients consulting primary care physicians in France. Fundam Clin Pharmacol. 2010;24:101–8. 19678853 10.1111/j.1472-8206.2009.00730.x

[37] Ekman M , Jönhagen S , Hunsche E , Jönsson L . Burden of illness of chronic low back pain in Sweden: a cross‐sectional, retrospective study in primary care setting. Spine (Phila Pa 1976). 2005;30:1777–85. 16094281 10.1097/01.brs.0000171911.99348.90

[38] Deyo RA , Von Korff M , Duhrkoop D . Opioids for low back pain. BMJ. 2015;350:g6380. 25561513 10.1136/bmj.g6380 PMC6882374

[39] Krebs EE , Gravely A , Nugent S , Jensen AC , DeRonne B , Goldsmith ES , et al. Effect of opioid vs nonopioid medications on pain‐related function in patients with chronic back pain or hip or knee osteoarthritis pain: the SPACE randomized clinical trial. JAMA. 2018;319:872–82. 29509867 10.1001/jama.2018.0899 PMC5885909

[40] Langley PC , Liedgens H . Time since diagnosis, treatment pathways and current pain status: a retrospective assessment in a back pain population. J Med Econ. 2013;16:701–9. 23425290 10.3111/13696998.2013.777346

[41] Conaghan PG , Doane MJ , Jaffe DH , et al. Are pain severity and current pharmacotherapies associated with quality of life, work productivity, and healthcare utilization for people with osteoarthritis in five large European countries?. Clin Exp Rheumatol. 2020;39:819–28. 32896256

[42] van Hout B , Janssen MF , Feng Y‐S , Kohlmann T , Busschbach J , Golicki D , et al. Interim scoring for the EQ‐5D‐5L: mapping the EQ‐5D‐5L to EQ‐5D‐3L value sets. Value Health. 2012;15:708–15. 22867780 10.1016/j.jval.2012.02.008

[43] National Institute for Health and Care Excellence . Position statement on use of the EQ‐5D‐5L value set for England (updated October 2019); 2019. https://www.nice.org.uk/about/what‐we‐do/our‐programmes/nice‐guidance/technology‐appraisal‐guidance/eq‐5d‐5l. Accessed 3 June 2020

[44] EuroQol Research Foundation . EQ‐5D‐5L valuation: standard value sets; 2020. https://euroqol.org/eq‐5d‐instruments/eq‐5d‐5l‐about/valuation‐standard‐value‐sets/. Accessed 3 June 2020

