# Supplementary material for: Burden of chronic low back pain: Association with pain severity and prescription medication use in five large European countries
Source: Pain Pract. 2021 Nov 26;22(3):359–71. doi: 10.1111/papr.13093 (PMC9298715; doi:10.1111/papr.13093)
Supplement: Supplementary file 1 — Table S1‐S5 [file PAPR-22-359-s001.docx]

**SUPPLEMENTARY INFORMATION**

**Table S1.** Germany

**Table S2.** France

**Table S3.** UK

**Table S4.** Italy

**Table S5.** Spain

**Table S1.** Health‑Related Outcomes, Work Productivity and Activity Impairment, and Healthcare Resource Utilization (Least Squares Mean ± SE) Among Respondents With Chronic Low Back Pain: Germany (*N =* 811)

|  | **Mild Pain  Rx-Untreated** | **Mild Pain  Rx-Treated** | **Moderate/Severe Pain Rx-Untreated** | **Moderate/Severe Pain Rx-Treated** |
| --- | --- | --- | --- | --- |
| **Health-related quality of life** |  |  |  |  |
| SF-12v2 PCS | 46.47 ± 0.92 | 39.13 ± 0.82* | 42.53 ± 0.66*^#^ | 34.90 ±0.72*^#†^ |
| SF-12v2 MCS | 40.28 ± 1.04 | 41.09 ± 0.94 | 39.28 ± 0.75 | 39.56 ± 0.82 |
| SF-12v2 Bodily Pain | 44.22 ± 0.91 | 35.89 ± 0.82* | 38.48 ± 0.65*^#^ | 31.12 ± 0.72*^#†^ |
| SF-12v2 General Health | 43.97 ± 0.93 | 38.37 ± 0.84* | 40.21 ± 0.67*^#^ | 35.53 ± 0.73*^#†^ |
| SF-12v2 Mental Health | 42.37 ± 1.01 | 41.08 ± 0.91 | 40.14 ± 0.72* | 39.24 ± 0.79*^#^ |
| SF-12v2 Physical Functioning | 47.00 ± 0.96 | 42.06 ± 0.87* | 44.31 ± 0.69*^#^ | 37.81 ± 0.76*^#†^ |
| SF-12v2 Role Emotional | 38.83 ± 1.17 | 38.20 ± 1.05 | 37.44 ± 0.84 | 35.22 ± 0.92*^#†^ |
| SF-12v2 Role Physical | 42.63 ± 0.93 | 38.11 ± 0.83* | 40.38 ± 0.67*^#^ | 34.82 ± 0.73*^#†^ |
| SF-12v2 Social Functioning | 42.40 ± 1.03 | 40.17 ± 0.93 | 39.97 ± 0.74* | 37.00 ± 0.81*^#†^ |
| SF-12v2 Vitality | 44.27 ± 0.96 | 43.07 ± 0.86 | 43.58 ± 0.69 | 41.46 ± 0.75*^†^ |
| **Health status** |  |  |  |  |
| SF-6D utility score | 0.64 ± 0.01 | 0.58 ± 0.01* | 0.60 ± 0.01* | 0.53 ± 0.01*^#†^ |
| EQ‑5D‑5L | 0.77 ± 0.02 | 0.69 ± 0.02* | 0.70 ± 0.02* | 0.54 ± 0.02*^#†^ |
| EQ VAS | 62.87 ± 2.18 | 50.41 ± 1.96* | 55.12 ± 1.57*^#^ | 42.41 ± 1.71*^#†^ |
| **WPAI-GH** |  |  |  |  |
| Absenteeism (%) | 6.65 ± 1.35 | 11.56 ± 2.37* | 12.19 ± 1.69* | 31.91 ± 5.11*^#†^ |
| Presenteeism (%) | 22.94 ± 4.24 | 44.02 ± 8.68* | 39.11 ± 5.59* | 57.87 ± 9.46*^†^ |
| Overall work impairment (%) | 24.68 ± 4.35 | 49.61 ± 9.30* | 45.29 ± 5.91* | 66.94 ± 10.02*^†^ |
| Activity impairment (%) | 32.03 ± 3.72 | 50.73 ± 5.22* | 47.16 ± 3.91* | 66.81 ± 6.06*^#†^ |
| **HRU (number of visits in last 6 months)** |  |  |  |  |
| Healthcare providers | 9.18 ± 1.12 | 13.23 ± 1.42* | 11.16 ± 0.97 | 17.99 ± 1.70*^#†^ |
| Primary care | 2.57 ± 0.36 | 4.08 ± 0.48* | 3.29 ± 0.32 | 5.19 ± 0.53*^#†^ |
| Emergency room or urgent care | 0.10 ± 0.04 | 0.29 ± 0.07* | 0.18 ± 0.04 | 0.33 ± 0.07*^†^ |
| Hospitalization | 0.18 ± 0.06 | 0.18 ± 0.05 | 0.17 ± 0.04 | 0.27 ± 0.06^†^ |

*Differs from mild pain Rx-untreated, *p* ≤ 0.05.

^#^Differs from mild pain Rx-treated, *p* ≤ 0.05.

^†^Differs from moderate/severe pain Rx-untreated, *p* ≤ 0.05.

Generalized linear models specifying a normal distribution and identity function were used with normally distributed outcome variables whereas models specifying a negative binomial distribution and log-link function were used with highly positively skewed outcome variables such as work productivity and activity impairment. Covariates included: severity/treatment group, country of residence, age, sex, marital status, education, income, employment status, alcohol use, exercise, body mass index, smoking status, anxiety diagnosis, depression diagnosis, insomnia diagnosis, diagnosed with sleep difficulties, and CCI. Least squares mean values for individual countries calculated using an interaction term of group by country in the overall model. EQ-5D-5L based on country-specific value set.

CCI, Charlson Comorbidity Index; EQ VAS, EQ visual analogue scale; HRU, healthcare resource utilization; MCS, mental component summary score; PCS, physical component summary score; Rx, prescription medication; SE, standard error; SF-12v2, Medical Outcomes Study 12-Item Short Form Survey Instrument version 2; SF-6D, Short Form-6 Dimensions; WPAI-GH, Work Productivity and Activity Impairment-General Health.

**Table S2.** Health‑Related Outcomes, Work Productivity and Activity Impairment, and Healthcare Resource Utilization (Least Squares Mean ± SE) Among Respondents With Chronic Low Back Pain: France (*N =* 520)

|  | **Mild Pain  Rx-Untreated** | **Mild Pain  Rx-Treated** | **Moderate/Severe Pain Rx-Untreated** | **Moderate/Severe Pain Rx-Treated** |
| --- | --- | --- | --- | --- |
| **Health-related quality of life** |  |  |  |  |
| SF-12v2 PCS | 46.56 ± 1.39 | 40.61 ± 0.96* | 44.54 ± 0.74^#^ | 38.71 ± 0.78*^†^ |
| SF-12v2 MCS | 41.08 ± 1.57 | 38.87 ± 1.08 | 38.47 ± 0.84 | 35.18 ± 0.89*^#†^ |
| SF-12v2 Bodily Pain | 43.27 ± 1.38 | 36.96 ± 0.95* | 40.60 ± 0.74^#^ | 33.24 ± 0.78*^#†^ |
| SF-12v2 General Health | 44.16 ± 1.41 | 41.34 ± 0.97 | 43.17 ± 0.75 | 38.55 ± 0.80*^#†^ |
| SF-12v2 Mental Health | 43.22 ± 1.52 | 40.85 ± 1.05 | 40.90 ± 0.81 | 37.19 ± 0.86*^#†^ |
| SF-12v2 Physical Functioning | 48.17 ± 1.46 | 41.70 ± 1.00* | 45.32 ± 0.78^#^ | 39.66 ± 0.82*^†^ |
| SF-12v2 Role Emotional | 41.01 ± 1.76 | 35.91 ± 1.21* | 37.46 ± 0.94* | 31.91 ± 1.00*^#†^ |
| SF-12v2 Role Physical | 43.96 ± 1.40 | 38.31 ± 0.96* | 41.50 ± 0.75^#^ | 36.38 ± 0.79*^†^ |
| SF-12v2 Social Functioning | 42.01 ± 1.55 | 37.30 ± 1.07* | 39.42 ± 0.83 | 34.82 ± 0.88*^#†^ |
| SF-12v2 Vitality | 43.82 ± 1.44 | 42.01 ± 1.00 | 41.77 ± 0.77 | 39.37 ± 0.82*^#†^ |
| **Health status** |  |  |  |  |
| SF-6D utility score | 0.65 ± 0.02 | 0.57 ± 0.01* | 0.60 ± 0.01*^#^ | 0.54 ± 0.01*^#†^ |
| EQ‑5D‑5L | 0.65 ± 0.03 | 0.47 ± 0.02* | 0.60 ± 0.02^#^ | 0.37 ± 0.02*^#†^ |
| EQ VAS | 62.72 ± 3.30 | 52.00 ± 2.27* | 56.62 ± 1.76^#^ | 44.34 ± 1.87*^#†^ |
| **WPAI-GH** |  |  |  |  |
| Absenteeism (%) | 6.89 ± 2.23 | 15.85 ± 3.57* | 1.44 ± 0.31*^#^ | 23.16 ± 4.28*^†^ |
| Presenteeism (%) | 20.81 ± 6.56 | 37.74 ± 8.60 | 28.69 ± 4.96 | 40.71 ± 8.05* |
| Overall work impairment (%) | 27.05 ± 8.03 | 50.31 ± 10.58 | 29.29 ± 4.84^#^ | 55.88 ± 9.71*^†^ |
| Activity impairment (%) | 28.51 ± 5.02 | 56.15 ± 6.79* | 43.77 ± 4.08* | 64.60 ± 6.34*^†^ |
| **HRU (number of visits in last 6 months)** |  |  |  |  |
| Healthcare providers | 7.66 ± 1.45 | 12.77 ± 1.61* | 9.76 ± 0.97 | 13.10 ± 1.35*^†^ |
| Primary care | 2.20 ± 0.48 | 3.97 ± 0.54* | 2.88 ± 0.32^#^ | 4.43 ± 0.50*^†^ |
| Emergency room or urgent care | 0.07 ± 0.05 | 0.17 ± 0.06 | 0.18 ± 0.05 | 0.20 ± 0.05 |
| Hospitalization | 0.63 ± 0.22 | 0.31 ± 0.09 | 0.17 ± 0.05* | 0.32 ± 0.08^†^ |

*Differs from mild pain Rx-untreated, *p* ≤ 0.05.

#Differs from mild pain Rx-treated, *p* ≤ 0.05.

†Differs from moderate/severe pain Rx-untreated, *p* ≤ 0.05.

Generalized linear models specifying a normal distribution and identity function were used with normally distributed outcome variables whereas models specifying a negative binomial distribution and log-link function were used with highly positively skewed outcome variables such as work productivity and activity impairment. Covariates included: severity/treatment group, country of residence, age, sex, marital status, education, income, employment status, alcohol use, exercise, body mass index, smoking status, anxiety diagnosis, depression diagnosis, insomnia diagnosis, diagnosed with sleep difficulties, and CCI. Least squares mean values for individual countries calculated using an interaction term of group by country in the overall model. EQ-5D-5L based on country-specific value set.

CCI, Charlson Comorbidity Index; EQ VAS, EQ Visual Analogue Scale; HRU, healthcare resource utilization; MCS, mental component summary score; PCS, physical component summary score; Rx, prescription medication; SE, standard error; SF-12v2, Medical Outcomes Study 12-Item Short Form Survey Instrument version 2; SF-6D, Short Form-6 Dimensions; WPAI-GH, Work Productivity and Activity Impairment-General Health.

**Table S3.** Health‑Related Outcomes, Work Productivity and Activity Impairment, and Healthcare Resource Utilization (Least Squares Mean ± SE) Among Respondents With Chronic Low Back Pain: UK (*N =* 388)

|  | **Mild Pain  Rx-Untreated** | **Mild Pain  Rx-Treated** | **Moderate/Severe Pain Rx-Untreated** | **Moderate/Severe Pain Rx-Treated** |
| --- | --- | --- | --- | --- |
| **Health-related quality of life** |  |  |  |  |
| SF-12v2 PCS | 47.65 ± 1.09 | 38.77 ± 1.02* | 42.69 ± 0.87*^#^ | 33.68 ± 0.84*^#†^ |
| SF-12v2 MCS | 40.69 ± 1.23 | 42.22 ± 1.16 | 40.12 ± 0.99 | 37.60 ± 0.95*^#†^ |
| SF-12v2 Bodily Pain | 45.20 ± 1.08 | 37.40 ± 1.01* | 40.98 ± 0.87*^#^ | 31.50 ± 0.83*^#†^ |
| SF-12v2 General Health | 44.13 ± 1.10 | 38.25 ± 1.04* | 39.47 ± 0.89* | 32.71 ± 0.85*^#†^ |
| SF-12v2 Mental Health | 41.44 ± 1.19 | 42.06 ± 1.12 | 40.08 ± 0.96 | 37.69 ± 0.92*^#†^ |
| SF-12v2 Physical Functioning | 46.95 ± 1.14 | 40.89 ± 1.07* | 43.23 ± 0.92* | 35.47 ± 0.88*^#†^ |
| SF-12v2 Role Emotional | 41.70 ± 1.38 | 40.80 ± 1.29 | 40.42 ± 1.11 | 35.14 ± 1.06*^#†^ |
| SF-12v2 Role Physical | 45.62 ± 1.09 | 39.29 ± 1.03* | 41.80 ± 0.88*^#^ | 34.75 ± 0.85*^#†^ |
| SF-12v2 Social Functioning | 44.82 ± 1.22 | 39.93 ± 1.14* | 41.46 ± 0.98* | 35.29 ± 0.94*^#†^ |
| SF-12v2 Vitality | 42.23 ± 1.13 | 41.40 ± 1.06 | 40.87 ± 0.91 | 36.27 ± 0.87*^#†^ |
| **Health status** |  |  |  |  |
| SF-6D utility score | 0.68 ± 0.01 | 0.60 ± 0.01* | 0.62 ± 0.01* | 0.53 ± 0.01*^#†^ |
| EQ‑5D‑5L | 0.66 ± 0.03 | 0.54 ± 0.02* | 0.58 ± 0.02* | 0.36 ± 0.02*^#†^ |
| EQ VAS | 64.31 ± 2.59 | 51.92 ± 2.42* | 53.60 ± 2.08* | 38.86 ± 2.00*^#†^ |
| **WPAI-GH** |  |  |  |  |
| Absenteeism (%) | 3.49 ± 0.90 | 18.27 ± 4.25* | 12.35 ± 2.80* | 25.36 ± 5.79*^†^ |
| Presenteeism (%) | 16.85 ± 3.82 | 40.69 ± 9.71* | 33.94 ± 7.35* | 50.43 ± 11.97* |
| Overall work impairment (%) | 21.14 ± 4.57 | 50.97 ± 11.40* | 41.92 ± 8.67* | 61.34 ± 13.34* |
| Activity impairment (%) | 28.73 ± 3.96 | 49.76 ± 6.38* | 43.63 ± 4.80* | 62.49 ± 6.62*^†^ |
| **HRU (number of visits in last 6 months)** |  |  |  |  |
| Healthcare providers | 5.80 ± 0.88 | 8.41 ± 1.15* | 5.83 ± 0.69^#^ | 9.09 ± 1.02*^†^ |
| Primary care | 1.99 ± 0.34 | 3.43 ± 0.51* | 1.87 ± 0.26^#^ | 3.41 ± 0.41*^†^ |
| Emergency room or urgent care | 0.06 ± 0.04 | 0.38 ± 0.11* | 0.15 ± 0.05^#^ | 0.37 ± 0.09*^†^ |
| Hospitalization | 0.05 ± 0.03 | 0.16 ± 0.05 | 0.11 ± 0.04 | 0.19 ± 0.05* |

*Differs from mild pain Rx-untreated, *p* ≤ 0.05.

#Differs from mild pain Rx-treated, *p* ≤ 0.05.

†Differs from moderate/severe pain Rx-untreated, *p* ≤ 0.05.

Generalized linear models specifying a normal distribution and identity function were used with normally distributed outcome variables whereas models specifying a negative binomial distribution and log-link function were used with highly positively skewed outcome variables such as work productivity and activity impairment. Covariates included: severity/treatment group, country of residence, age, sex, marital status, education, income, employment status, alcohol use, exercise, body mass index, smoking status, anxiety diagnosis, depression diagnosis, insomnia diagnosis, diagnosed with sleep difficulties, and CCI. Least squares mean values for individual countries calculated using an interaction term of group by country in the overall model. EQ-5D-5L based on country-specific value set.

CCI, Charlson Comorbidity Index; EQ VAS, EQ visual analogue scale; HRU, healthcare resource utilization; MCS, mental component summary score; PCS, physical component summary score; Rx, prescription medication; SE, standard error; SF-12v2, Medical Outcomes Study 12-Item Short Form Survey Instrument version 2; SF-6D, Short Form-6 Dimensions; WPAI-GH, Work Productivity and Activity Impairment-General Health.

**Table S4.** Health‑Related Outcomes, Work Productivity and Activity Impairment, and Healthcare Resource Utilization (Least Squares Mean ± SE) Among Respondents With Chronic Low Back Pain: Italy (*N =* 192)

|  | **Mild Pain  Rx-Untreated** | **Mild Pain  Rx-Treated** | **Moderate/Severe Pain Rx-Untreated** | **Moderate/Severe Pain Rx-Treated** |
| --- | --- | --- | --- | --- |
| **Health-related quality of life** |  |  |  |  |
| SF-12v2 PCS | 46.22 ± 1.62 | 41.29 ± 1.52* | 45.31 ± 0.96^#^ | 39.80 ± 1.52*^†^ |
| SF-12v2 MCS | 36.47 ± 1.84 | 39.77 ± 1.73 | 36.60 ± 1.09 | 38.85 ± 1.73 |
| SF-12v2 Bodily Pain | 41.06 ± 1.61 | 36.38 ± 1.51* | 39.33 ± 0.96 | 34.45 ± 1.51*^†^ |
| SF-12v2 General Health | 43.36 ± 1.65 | 39.97 ± 1.55 | 41.10 ± 0.98 | 37.66 ± 1.55*^†^ |
| SF-12v2 Mental Health | 39.79 ± 1.78 | 42.21 ± 1.67 | 39.22 ± 1.06 | 41.38 ± 1.67 |
| SF-12v2 Physical Functioning | 47.18 ± 1.71 | 44.59 ± 1.60 | 46.69 ± 1.01 | 42.85 ± 1.60*^†^ |
| SF-12v2 Role Emotional | 36.23 ± 2.06 | 35.87 ± 1.93 | 35.27 ± 1.22 | 35.57 ± 1.93 |
| SF-12v2 Role Physical | 41.75 ± 1.64 | 38.70 ± 1.53 | 42.04 ± 0.97^#^ | 39.16 ± 1.54 |
| SF-12v2 Social Functioning | 36.43 ± 1.82 | 37.86 ± 1.70 | 38.18 ± 1.08 | 34.11 ± 1.71^†^ |
| SF-12v2 Vitality | 43.24 ± 1.69 | 45.01 ± 1.58 | 43.55 ± 1.01 | 45.26 ± 1.59 |
| **Health status** |  |  |  |  |
| SF-6D utility score | 0.58 ± 0.02 | 0.59 ± 0.02 | 0.58 ± 0.01 | 0.55 ± 0.02 |
| EQ‑5D‑5L | 0.75 ± 0.04 | 0.70 ± 0.04 | 0.74 ± 0.02 | 0.68 ± 0.04 |
| EQ VAS | 59.84 ± 3.86 | 50.91 ± 3.62 | 60.70 ± 2.29^#^ | 48.13 ± 3.62*^†^ |
| **WPAI-GH** |  |  |  |  |
| Absenteeism (%) | 4.18 ± 1.28 | 37.32 ± 10.75* | 9.45 ± 2.06*^#^ | 16.32 ± 5.59* |
| Presenteeism (%) | 34.43 ± 9.76 | 38.89 ± 11.10 | 44.45 ± 9.19 | 27.14 ± 9.16 |
| Overall work impairment (%) | 36.47 ± 9.87 | 52.25 ± 14.03 | 48.22 ± 9.54 | 37.38 ± 12.02 |
| Activity impairment (%) | 38.24 ± 7.83 | 64.16± 12.24 | 45.79 ± 5.55 | 60.13 ± 11.54 |
| **HRU (number of visits in last 6 months)** |  |  |  |  |
| Healthcare providers | 10.32 ± 2.25 | 12.40 ± 2.48 | 9.77 ± 1.26 | 14.89 ± 3.00 |
| Primary care | 4.62 ± 1.07 | 5.14 ± 1.09 | 3.89 ± 0.55 | 6.08± 1.29 |
| Emergency room or urgent care | 0.04 ± 0.04 | 0.39 ± 0.15* | 0.31 ± 0.09* | 0.24 ± 0.11 |
| Hospitalization | 0.00 ± 0.00 | 0.13 ± 0.08 | 0.12 ± 0.05* | 0.13 ± 0.08 |

*Differs from mild pain Rx-untreated, *p* ≤ 0.05.

#Differs from mild pain Rx-treated, *p* ≤ 0.05.

†Differs from moderate/severe pain Rx-untreated, *p* ≤ 0.05.

Generalized linear models specifying a normal distribution and identity function were used with normally distributed outcome variables whereas models specifying a negative binomial distribution and log-link function were used with highly positively skewed outcome variables such as work productivity and activity impairment. Covariates included: severity/treatment group, country of residence, age, sex, marital status, education, income, employment status, alcohol use, exercise, body mass index, smoking status, anxiety diagnosis, depression diagnosis, insomnia diagnosis, diagnosed with sleep difficulties, and CCI. Least squares mean values for individual countries calculated using an interaction term of group by country in the overall model. EQ-5D-5L based on country-specific value set.

CCI, Charlson Comorbidity Index; EQ VAS, EQ visual analogue scale; HRU, healthcare resource utilization; MCS, mental component summary score; PCS, physical component summary score; Rx, prescription medication; SE, standard error; SF-12v2, Medical Outcomes Study 12-Item Short Form Survey Instrument version 2; SF-6D, Short Form-6 Dimensions; WPAI-GH, Work Productivity and Activity Impairment-General Health.

**Table S5.** Health‑Related Outcomes, Work Productivity and Activity Impairment, and Healthcare Resource Utilization (Least Squares Mean ± SE) Among Respondents With Chronic Low Back Pain: Spain (*N =* 157)

|  | **Mild Pain  Rx-Untreated** | **Mild Pain  Rx-Treated** | **Moderate/Severe Pain Rx-Untreated** | **Moderate/Severe Pain Rx-Treated** |
| --- | --- | --- | --- | --- |
| **Health-related quality of life** |  |  |  |  |
| SF-12v2 PCS | 50.18 ± 1.80 | 43.50 ± 1.26* | 43.48 ± 1.32* | 40.24 ± 1.33* |
| SF-12v2 MCS | 45.03 ± 2.04 | 39.89 ± 1.43* | 40.03 ± 1.50* | 40.12 ± 1.50* |
| SF-12v2 Bodily Pain | 50.35 ± 1.78 | 40.90 ± 1.25* | 40.81 ± 1.31* | 36.60 ± 1.32*^#†^ |
| SF-12v2 General Health | 45.44 ± 1.83 | 40.27 ± 1.28* | 39.10 ± 1.34* | 38.52 ± 1.35* |
| SF-12v2 Mental Health | 46.26 ± 1.97 | 41.66 ± 1.38* | 40.86 ± 1.45* | 41.07 ± 1.45* |
| SF-12v2 Physical Functioning | 49.69 ± 1.89 | 44.03 ± 1.32* | 44.56 ± 1.39* | 40.87 ± 1.39*^†^ |
| SF-12v2 Role Emotional | 43.49 ± 2.28 | 36.62 ± 1.60* | 38.13 ± 1.68* | 36.06 ± 1.68* |
| SF-12v2 Role Physical | 48.01 ± 1.81 | 41.49 ± 1.27* | 42.20 ± 1.33* | 40.72 ± 1.34* |
| SF-12v2 Social Functioning | 47.75 ± 2.02 | 39.57± 1.41* | 41.22 ± 1.48* | 39.68 ± 1.49* |
| SF-12v2 Vitality | 49.82 ± 1.87 | 46.29 ± 1.31 | 44.62 ± 1.38* | 44.02 ± 1.38* |
| **Health status** |  |  |  |  |
| SF-6D utility score | 0.71 ± 0.02 | 0.61 ± 0.02* | 0.60 ± 0.02* | 0.59 ± 0.02* |
| EQ‑5D‑5L | 0.80 ± 0.04 | 0.68 ± 0.03* | 0.68 ± 0.03* | 0.57 ± 0.03*^#†^ |
| EQ VAS | 70.92 ± 4.28 | 60.84 ± 2.99* | 59.25 ± 3.14* | 45.82 ± 3.15*^#†^ |
| **WPAI-GH** |  |  |  |  |
| Absenteeism (%) | 1.22 ± 0.49 | 13.92 ± 3.80* | 12.12 ± 3.48* | 25.52 ± 8.41* |
| Presenteeism (%) | 20.40 ± 6.28 | 32.73 ± 8.44 | 34.32 ± 9.00 | 52.93 ± 16.87 |
| Overall work impairment (%) | 19.79 ± 6.01 | 38.42 ± 9.63 | 38.08 ± 9.86 | 54.45 ± 16.62* |
| Activity impairment (%) | 22.05 ± 5.06 | 41.12 ± 6.55* | 43.08 ± 7.19* | 53.91 ± 9.01* |
| **HRU (number of visits in last 6 months)** |  |  |  |  |
| Healthcare providers | 7.29 ± 1.80 | 9.28 ± 1.56 | 8.98 ± 1.60 | 11.04 ± 1.93 |
| Primary care | 2.46 ± 0.68 | 2.91 ± 0.54 | 3.06 ± 0.60 | 3.67 ± 0.70 |
| Emergency room or urgent care | 0.31 ± 0.17 | 0.54 ± 0.16 | 0.47 ± 0.15 | 1.06 ± 0.29*^†^ |
| Hospitalization | 0.00 ± 0.00 | 0.13 ± 0.06* | 0.09 ± 0.05 | 0.19 ± 0.08* |

*Differs from mild pain Rx-untreated, *p* ≤ 0.05.

#Differs from mild pain Rx-treated, *p* ≤ 0.05.

†Differs from moderate/severe pain Rx-untreated, *p* ≤ 0.05.

Generalized linear models specifying a normal distribution and identity function were used with normally distributed outcome variables whereas models specifying a negative binomial distribution and log-link function were used with highly positively skewed outcome variables such as work productivity and activity impairment. Covariates included: severity/treatment group, country of residence, age, sex, marital status, education, income, employment status, alcohol use, exercise, body mass index, smoking status, anxiety diagnosis, depression diagnosis, insomnia diagnosis, diagnosed with sleep difficulties, and CCI. Least squares mean values for individual countries calculated using an interaction term of group by country in the overall model. EQ-5D-5L based on country-specific value set.

CCI, Charlson Comorbidity Index; EQ VAS, EQ visual analogue scale; HRU, healthcare resource utilization; MCS, mental component summary score; PCS, physical component summary score; Rx, prescription medication; SE, standard error; SF-12v2, Medical Outcomes Study 12-Item Short Form Survey Instrument version 2; SF-6D, Short Form-6 Dimensions; WPAI-GH, Work Productivity and Activity Impairment-General Health.
